# Prevalence and Risk Factors of Hookworm-Related Cutaneous Larva Migrans (HrCLM) in a Resource-Poor Community in Manaus, Brazil

**DOI:** 10.1371/journal.pntd.0004514

**Published:** 2016-03-24

**Authors:** Felix Reichert, Daniel Pilger, Angela Schuster, Hannah Lesshafft, Silas Guedes de Oliveira, Ralf Ignatius, Hermann Feldmeier

**Affiliations:** 1 Institute of Microbiology and Hygiene, Charité University Medical Center, Berlin, Germany; 2 Department of Neonatology, Charité University Medical Center, Berlin, Germany; 3 Department of Ophtalmology, Charité University Medical Center, Berlin, Germany; 4 London School of Hygiene & Tropical Medicine, London, United Kingdom; 5 School of Social and Political Science, University of Edinburgh, Edinburgh, United Kingdom; 6 Foundation for Tropical Medicine of Amazonas (FMTAM), Manaus, Amazonas, Brazil; 7 Hematology and Hemotherapy Foundation from Amazonas State (HEMOAM), Manaus, Amazonas, Brazil; 8 Labor Enders, Stuttgart, Germany; The George Washington University School of Medicine and Health Sciences, UNITED STATES

## Abstract

**Background:**

Hookworm-related cutaneous larva migrans (HrCLM) is a neglected tropical skin disease associated with significant clinical pathology. Little knowledge exists about prevalence and risk factors of HrCLM in endemic regions.

**Methodology/ Principal Findings:**

To understand the epidemiology of HrCLM in Amazonia, we conducted a cross-sectional study in a resource-poor township in Manaus, Brazil. HrCLM was diagnosed in 8.2% (95% CI, 6.3–10.1%) of the study population (N = 806) with a peak prevalence of 18.2% (95% CI, 9.3–27.1%) in children aged 10–14. Most of the tracks (62.4%) were located on the feet, and 10.6% were superinfected. HrCLM was associated independently with age under 15, male sex, presence of animal faeces on the compound, walking barefoot on sandy ground and poverty.

**Conclusions/ Significance:**

HrCLM is common in resource-poor communities in Amazonia and is related to poverty. To reduce the disease burden caused by HrCLM, living conditions have to be improved.

## Author Summary

Hookworm-related cutaneous larva migrans (HrCLM) is a parasitic skin disease caused by the penetration of animal hookworm larvae into the human skin. In this compartment the larvae cannot pass the basal membrane and reproduce, but migrate in the outer skin layer for several weeks, causing skin inflammation and intense itching. Thus, humans are a biological impasse. Although HrCLM is a common skin disease in tropical and subtropical regions, studies on prevalence and risk factors are scarce. We clinically examined the population of a resource-poor neighbourhood in Manaus, capital of Amazonas State, Brazil, and investigated HrCLM-associated risk factors. HrCLM was very common with an overall prevalence of 8.2%. Children in general, and boys in particular, were most frequently infected. We could confirm that walking barefoot on sandy ground is a significant risk factor, and we identified the presence of animal faeces on the compound as another important predictive factor. Clearly, HrCLM was associated with low income and poverty-related living conditions. The poorest of the poor were identified as the most vulnerable population group.

## Introduction

Hookworm-related cutaneous larva migrans (HrCLM) is a parasitic skin disease caused by the penetration of feline or canine hookworm larvae into the human epidermis. The most frequent species are *Ancylostoma braziliense*, *Ancylostoma caninum* and *Uncinaria stenocephal*a [1–3]. In humans, the larva is unable to cross the basal membrane of the epidermis and migrates in the compartment of the epidermis until it dies spontaneously after a few weeks to several months [1,4,5]. The migration of animal hookworm larvae causes a typical elevated erythematous linear or serpiginous track known as “creeping eruption” [6]. HrCLM is associated with intense pruritus and significantly impairs the quality of life [7]. The resulting scratching leads to denudation of the skin, which facilitates bacterial superinfection of the lesion [1,8,9]. Additional skin injury may be caused by inappropriate surgical manipulation of the lesion and treatment with toxic substances [10].

Whereas animal hookworm species parasitize dogs and cats worldwide [11], HrCLM is mainly seen in tropical and subtropical areas in South America, the Caribbean, Africa and South-East Asia [11–14]. Sporadic cases have been reported for Europe [15–20]. In semi-arid north-eastern Brazil, prevalence ranged from 0.2% to 4.4% in the general population and from 0% to 14.9% in children <5 years [21–23]. No population based data exists for other endemic areas.

Known risk factors are male sex, young age, living in a house without a solid floor and barefoot walking [8,23]. An association with low income has been suspected [23].

In order to investigate the epidemiology of HrCLM in Amazonia and to develop sustainable means of control, in a first step we determined prevalence and risk factors in a resource-poor community in the outskirts of Manaus. Data of a spatial analysis will be published separately.

## Methods

### Study area and population

The study was conducted in Manaus, capital of Amazonas State, North Brazil. Manaus is situated at 03°06' south latitude and has a hot humid climate. The average annual precipitation is 2307mm and the mean annual temperature is 26.7°C (International Institute of Meteorology of Brazil, http://www.inmet.gov.br/portal/index.php?r=clima/normaisclimatologicas).

The study area is part of Nova Vitoria, a resource-poor neighbourhood at the outskirts of Manaus. The boundaries of the study area are defined on three sides by an *igarapé*, a small affluent of the Amazon River. On the fourth side a paved road separates the study area from Grande Vitoria, another resource-poor community. The study area is characterized by unpaved roads, absence of public health facilities, kindergartens or public schools. There was no sewage disposal system at the time of the study. Electricity was available but only half of the households were legally connected to the grid; the other half used hand-made wire connections. Drinking water was distributed via rubber hoses, which often flooded the streets. Many cats and dogs strayed around in the streets and gardens. Children usually played on the compound of the house, in the streets or on improvised football fields. Hence, the study area was representative for the innumerable poor neighbourhoods at the periphery of Manaus.

### Study design

As a first step into a comprehensive series of investigations on the epidemiology of HrCLM in Amazonia, we conducted a cross-sectional study in Nova Vitoria in April 2009, at the end of the rainy season. First, a census of all households and inhabitants was performed. During a door-to-door survey, households were GPS-mapped and environmental, socio-economic and behaviour-related risk factors were documented using a pre-tested, structured questionnaire. Inclusion criteria were residency in the study area for more than two months and provision of an informed, written consent.

All participants were examined clinically for HrCLM. The examination took place in the house where the family lived, in a room where privacy was guaranteed. The genital area was spared in case of absence of symptoms such as itching. HrCLM was diagnosed clinically by two investigators (DP and FR) when the characteristic slow-moving, elevated linear or serpiginous tracks were present [1,6,7,11–13,24]. Lesions were counted and the appearance and location of the tracks were documented. Each track was defined as a single lesion. Bacterial superinfection was diagnosed when pustules or suppuration were visible.

### Ethical considerations

The study was approved by the Ethical Committee of the Fundação de Medicina Tropical- Amazonas (FMT-AM). Informed, written consent was obtained from each participant or in the case of minors from their legal guardian. Each affected inhabitant of Nova Vitoria was offered free treatment independently of the participation in the study. Treatment consisted of ivermectin (Ivermec, Uci-farma, São Paulo, Brazil) given as single oral dose (200 μg/kg) or—in the case of children <5 years or <15 kg and women with suspected or confirmed pregnancy—of topically applied thiabendazole (5%; Tiadol, Bunker Indústria Farmacêutica Ltda., São Paulo, Brazil) 3 times a day for one week.

### Statistics

Data were entered in Microsoft Office Access 2007, cleaned for entering errors and analysed using PASW Statistics Version 18.0 (SPSS Inc., Chicago, USA). Missing data were assumed to be missing at random and flagged up in the analysis. Only complete cases were analysed.

An asset index was formed using principal component analysis (PCA) to categorize households according to socio-economic status. First, a set of assets that reflect wealth was identified. From this set of assets, we selected items with a high inequity in distribution among the households and a high eigenvalue [25]. Included assets were presence of a car, television, fridge, type of house construction, legal connection to electricity and monthly mobile phone costs. Using these assets, an index (“wealth score”) was built based on the respective value of each item in the PCA [25]. Households were ranked and divided into tertiles representing a high, intermediate or low socio-economic status. Income was categorized into three categories with the official minimum wage (R$ 465 per month in 2009) as a reference.

A knowledge score was derived out of six questions concerning the etiology of HrCLM. Every correct answer added one point to the score. The knowledge score values were categorized in tertiles representing households with little knowledge (0–3 correct answers), moderate knowledge (4 correct answers) and high knowledge (5–6 correct answers). Age groups were formed similar to previous population-based studies on HrCLM to allow comparison of the results [8,21,23].

For bivariable risk factor analysis, odds ratios (OR) were calculated together with 95% confidence intervals (95% CI). Statistical analysis consisted of χ²-test or Fisher-exact-test to compare relative frequencies and logistic regression for non-binary variables.

For multivariable risk factor analysis, all variables that showed weak evidence of an association with HrCLM (p<0.1) were entered into a stepwise logistic regression. We observed standard errors and 95% CI to identify multicollinearity and removed variables where necessary. A random effects model was used to control for clustering on household level.

## Results

According to the census 412 households existed in the study area, 127 of which were found without a resident present. Of the remaining 285 households, 5 (2%) did not match the inclusion criteria and 18 (6%) refused to participate. The remaining 262 households (92%) were inhabited by a total of 1104 people out of whom 806 (73%) were present during sampling and were included in the study.

Seventy-eight per cent of the adults were unemployed or working in the informal sector. Fifty-eight per cent of the households had one minimum wage (R$ 465 per month) or less at their disposition. The proportion of illiteracy in adults was at least 27%. Only 11.5% of the households had been visited by a community health worker within the last 12 months. Thirty-one per cent of the households stated that at least one case of HrCLM had occurred in household members within the last 12 months. (Table 1)

**Table 1:** Demographic, socio-economic and environmental characteristics of study households (N = 262).

Characteristic	n	%
**Demography**		
*Persons per household*: *median (range)*	4 (1–11)	
*Children per household*: *median (range)*	2 (0–8)	
**Economy**		
*Monthly per capita-income in R$*: median (range)*	116 (0–1500)	
Monthly income per household*		
< 1 minimum wage†	74	28.2
1 minimum wage†	78	29.8
> 1 minimum wage†	107	40.8
Reported food shortage experienced in the last 12 months*		
Yes	82	31.3
No	179	68.3
**Education**		
*Highest educational level in the household*		
Secondary school or higher	85	32.4
Only primary school	122	46.6
No education at all	55	21.0
*Number of households with ≥ 1 child aged 6–15 not going to school*	27	22.0‡
Knowledge about HrCLM*§¶		
Little	84	32.1
Moderate	127	48.5
High	41	15.6
**House construction**		
*House constructed of*		
Plastered masonry	37	14.1
Non-plastered masonry	100	38.2
Wood and/or plastic foils	125	47.7
Floor made of*		
Sand or soil	16	6.1
Wood	19	7.3
Concrete or tiles	226	86.3
Compound not fenced in*	161	61.5
Compound fenced in with	93	35.5
Barbed wire	22	8.4
Paling	65	24.8
Bricks	6	2.3
**Animals**		
Household kept cat or dog*		
Yes	148	56.5
No	112	42.7
Stray cats or dogs on the compound*		
Yes	244	93.1
No	13	5.0
Presence of animal faeces on the compound*		
Yes	31	11.8
No	230	87.8

*Missing observations.

†Minimum wage in 2009: 465 R$ ≈ 220$.

‡ Percent of households with children aged 6–15.

§For definitions see methods.

¶Hookworm-related cutaneous larva migrans.

**Fig 1:**
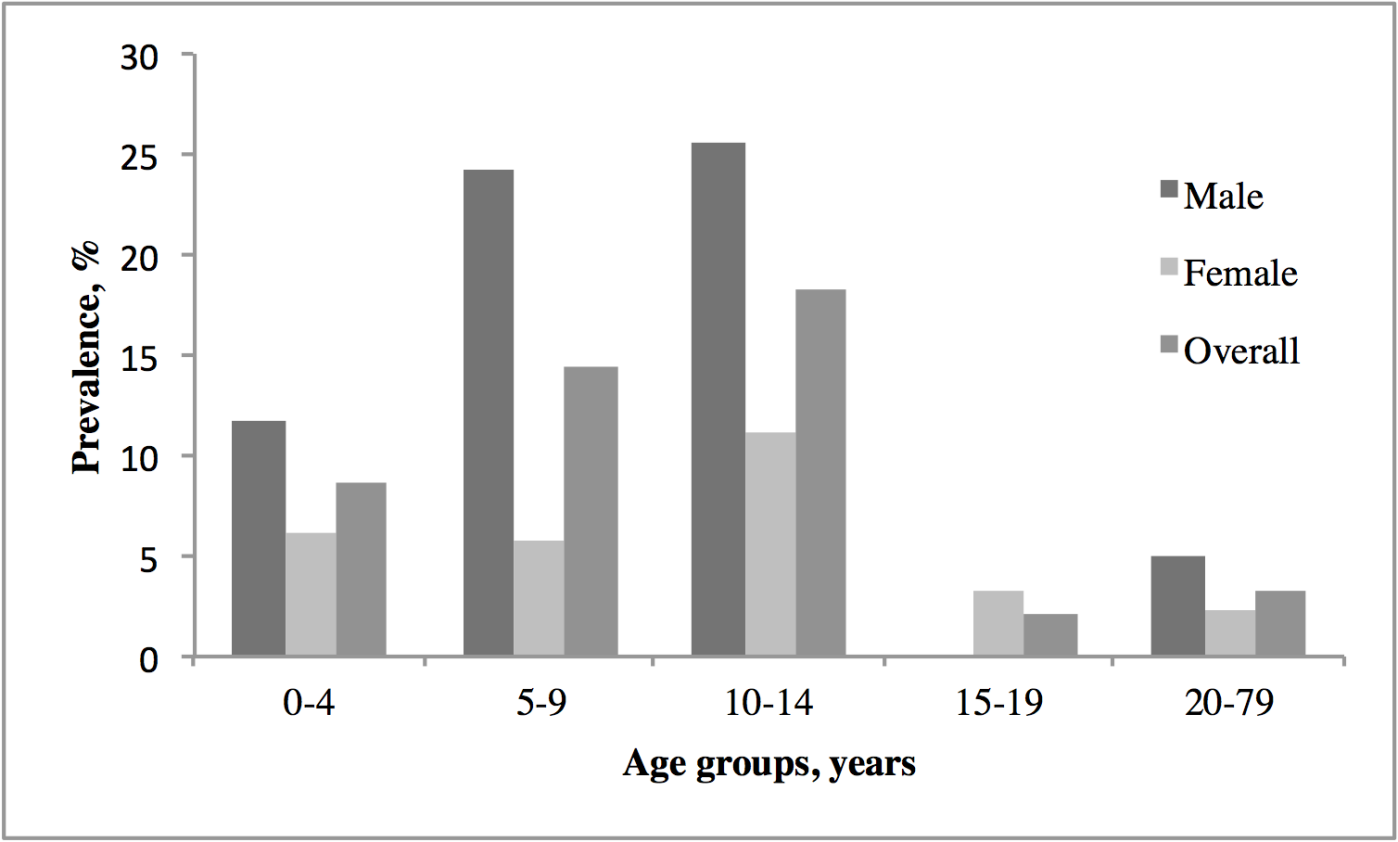
Prevalence of HrCLM (Hookworm-related cutaneous larva migrans) by age group and sex.

The median age was 13 years (range 0–72). The majority of the participants were females (59.3%). Sixty-six persons (8.2%; 95% CI, 6.3–10.1%) had HrCLM with a total of 117 lesions. Clinical characteristics of the infected study participants are presented in Table 2. Children aged 10–14 had the highest prevalence (18.2%; 95% CI, 9.3–27.1%; Fig 1). In all age groups of children, boys were significantly more affected than girls (p<0.001). The feet were the most common localisation of HrCLM.

Previous episodes of HrCLM were remembered of 18.7% of the participants. Following anamnestic information 39.7% had suffered of pediculosis capitis, 26.8% of tungiasis and 5.7% of scabies in the past year.

**Table 2:** Clinical characteristics of study participants with HrCLM (Hookworm-related cutaneous larva migrans) (N = 66).

Characteristic	n	%
Persons with superinfected lesions*	7	10.6
*Number of lesions per person*:		
1	36	54.5
2	21	31.8
≥3	9	13.6
*Topographic distribution of the lesions (n = 117)* †, ‡		
Foot	72	62.4§
Leg	9	7.7§
Trunk	9	7.7§
Arm	9	7.7§
Buttock	8	6.8§
Hand	6	5.1§
Head	1	0.9§

*Pustules or suppuration.

†Missing observations.

‡Multiple topographic affection occurred in 21.2% of persons with HrCLM.

§Percentage of all lesions (n = 117).

Bivariable risk factor analysis showed that male sex, age younger than 15, low family income, a low wealth score, playing football, practicing sport barefoot and presence of animal faeces on the compound were significantly associated with a high risk of HrCLM (Table 3). Those who reported to have had HrCLM in the last year had a significantly higher risk to be diagnosed with HrCLM in the cross-sectional study (OR = 15; 95% CI, 8.5–26.7). The highest risk was associated with the habit of always walking barefoot on sandy ground or soil (OR = 23.4; 95% CI, 8.0–68.6).

**Table 3:** Bivariable analysis (N = 806).

Characteristic	No.	HrCLM* (%)	Crude Odds Ratio (95% CI)	2-sided p-value
**Demography**				
*Male*	328	44 (13.4)	3.21 (1.89–5.47)	<0.001
Female	478	22 (4.6)	1 (reference)	
*Age*				
≤ 4 years	174	15 (8.6)	2.80 (1.26–6.25)	0.012
5–9 years	160	23 (14.4)	4.99 (2.37–10.52)	<0.001
10–14 years	88	16 (18.2)	6.61 (2.94–14.83)	<0.001
15–19 years	46	1 (2.2)	0.66 (0.08–5.24)	0.695
≥20 years	338	11 (3.3)	1 (reference)	
**Socioeconomic characteristics**				
*Income*				
< 1 minimum wage †, ‡	232	21 (9.1)	2.14 (1.05–4.38)	0.036
= 1 minimum wage‡	270	31 (11.5)	2.79 (1.43–5.46)	0.003
> 1 minimum wage‡	293	13 (4.4)	1 (reference)	
*Wealth score* †, §				
Low	321	37 (11.5)	3.16 (1.44–6.93)	0.004
Intermediate	263	21 (8.0)	2.10 (0.91–4.86)	0.081
High	202	8 (4.0)	1 (reference)	
**Education**				
*Knowledge about HrCLM* †, §				
Little	259	22 (8.5)	0.69 (0.35–1.36)	0.286
Moderate	384	25 (6.5)	0.52 (0.27–1.00)	0.051
High	135	16 (11.9)	1 (reference)	
**Behaviour**				
*Walking always/regularly* †				
Barefoot outdoor	58	14 (24.1)	4.16 (2.14–8.07)	<0.001
With sandals/shoes outdoor	731	52 (7.1)	1 (reference)	
*Walking on sandy ground* †				
Always barefoot	111	29 (26.1)	23.43 (8.00–68.60)	<0.001
Sometimes barefoot	420	33 (7.9)	5.65 (1.98–16.13)	0.001
Never barefoot	269	4 (1.5)	1 (reference)	
*Walking indoor*				
Walking barefoot and absence of solid floor †	103	15 (14.6)	2.18 (1.17–4.03)	0.019
Not walking barefoot or presence of solid floor	702	51 (7.3)	1 (reference)	
*Sports*				
Practicing football †	212	33 (15.6)	3.38 (1.99–5.76)	<0.001
Other sport	49	5 (10.2)	2.09 (0.77–5.67)	0.15
No sport	542	28 (5.2)	1 (reference)	
*Sport barefoot on sand* †	193	36 (18.7)	4.74 (1.41–15.95)	0.005
Sport never barefoot/not on sand	65	3 (4.6)	1 (reference)	
**Environment**				
*Animal faeces on compound* †	103	17 (16.5)	2.63 (1.45–4.78)	0.001
No faeces on compound	702	49 (7.0)	1 (reference)	
*Cat/dog ownership* †	469	36 (7.7)	0.99 (0.59–1.69)	0.983
No cat/dog ownership	324	25 (7.7)	1 (reference)	
*Stray cats/dogs on compound* †	753	65 (8.6)	3.78 (0.51–27.94)	0.243
No stray cats/dogs	41	*1 (2*.*4)*	1 (reference)	

*Hookworm-related cutaneous larva migrans.

†Missing observations.

‡Minimum wage in 2009: 465 R$ ≈ 220$.

§For definitions see Methods.

Multivariable risk factor analysis (Table 4) revealed that always walking barefoot on sandy ground or soil was the most important independent risk factor. Male sex, young age and presence of animal faeces on the compound remained independent risk factors for the presence of HrCLM. Obviously, HrCLM was significantly associated with poverty: A low wealth score of a household showed an adjusted odds ratio of 2.5 (95% CI, 1.1–5.8).

**Table 4:** Multivariable regression analysis.

Characteristic	Frequency (N = 779)	Adjusted odds ratio (95% CI)	2-sided p-value
*Sex*			
Male	319	2.30 (1.30–4.08)	0.004
Female	460	1 (reference)	
*Age*			
≤ 4 years	163	2.55 (1.11–5.90)	0.028
5–9 years	155	2.80 (1.26–6.23)	0.012
10–14 years	87	2.98 (1.23–7.21)	0.015
15–19 years	44	0.37 (0.05–3.03)	0.354
≥20 years	330	1 (reference)	
*Wealth score*			
Low	318	2.53 (1.10–5.82)	0.028
Intermediate	260	1.76 (0.73–4.22)	0.209
High	201	1 (reference)	
*Faeces found on compound*			
Yes	92	2.66 (1.34–5.29)	0.005
No	687	1 (reference)	
*Walking on sandy ground*			
Always barefoot	107	14.39 (4.62–44.85)	<0.001
Sometimes barefoot	406	4.76 (1.63–13.90)	0.004
Never barefoot	266	1 (reference)	

## Discussion

HrCLM is a neglected tropical disease associated with significant clinical pathology [26]. From a global perspective it is one of the most common parasitic skin diseases—and not primarily a health problem in returning travellers as publications in journals of travel medicine may make believe [11–13,27–29]. Only few epidemiological studies have been performed in endemic areas and population-based data exists exclusively from north-eastern Brazil. To understand the epidemiology of HrCLM in the Amazonas region, we conducted a cross-sectional study in the outskirts of Manaus and reported findings on prevalence, risk factors and clinical pathology.

### Clinical pathology

Clinical features were similar to those reported by others [12,13]. Most of the tracks (62.4%) were located on the feet, which reflects the fact that many people walked barefoot. This is consistent with our previous population-based study in rural Northeast Brazil [22]. The percentage of superinfected tracks was 10.6%. Previous studies in endemic areas by us and others reported similar proportions between 8 and 28% [8,21,22,30]. Unhygienic living conditions and practices as well as limited access to healthcare may explain the higher proportion of superinfected HrCLM in our study than usually seen in travellers [10,11].

### Prevalence

The overall prevalence of 8.2% (95% CI 6.3–10.1%) found in this study is the highest ever documented in a population-based study. Previous population-based studies in Northeast Brazil showed an overall prevalence between 0.2% and 4.4% during the dry and the raining season, respectively [8,21,23]. Similar to previous studies, the prevalence differed by age group and sex with a peak prevalence of 25.6% in 10–14 year old boys (Fig 1) [8,23]. Whether there is a seasonal variation in HrCLM prevalence in the Amazonas region, where the climate is hot and humid throughout the whole year, remains to be clarified. Outside Brazil only one prevalence study has been conducted on devotees of a temple in Sri Lanka. Fifty-eight per cent of the devotees were found to have HrCLM; however, it is doubtful whether this finding reflects the true overall prevalence in that area since the participants were examined after a special religious ritual increasing the odds for exposure [30].

The extremely high prevalence found in our study indicates excellent conditions for the completion of the off-host cycle of animal hookworm in Nova Vitoria. First, many stray dogs and cats roam in the community and act as animal reservoirs. There is no public veterinary service at all and pets are not treated against intestinal helminths. Animal faeces were present on 11.8% of all compounds, and faecal material littered many public areas. Second, hookworm eggs require an environment that protects them from desiccation to evolve into infective third stage larvae [31]. Manaus is located in the middle of the Amazon basin. The precipitation in the month preceding the study was around 230 mm with 20 days of rain (International Institute of Meteorology of Brazil (INMET)). All streets and most of the compounds in Nova Vitoria were unpaved and became muddy after heavy rainfall. Furthermore, the average temperature never falls below 25°C. This means that the environmental conditions are exceptionally favourable for the propagation of animal hookworm larvae [5]. And third, risky behaviour with prolonged contact to contaminated soil was frequent. Many children did not go to school but roamed through the streets and compounds the whole day, the majority walking barefoot at least part of the time.

### Risk factors

The multivariable model showed a complex pattern of risk factors with walking barefoot on sandy soil being most significant. This corroborates our previous findings from a semi-arid area of Brazil, where the lacking use of footwear was an independent risk factor [23]. For the first time we could show that the odds differed by the frequency protective footwear was used. Participants who always used shoes ran a lower risk of acquiring HrCLM than those wearing shoes sometimes (Table 4). Even the commonly used flip-flops (plastic sandals, which consist of a thin rubber sole with a single string) provided significant protection. However, closed shoes were worn regularly only by seven individuals.

Obviously, HrCLM was predominantly acquired outdoors. Neither walking barefoot indoors, even if the floor consisted of sand or soil, nor owning a cat or dog were identified as independent risk factors. Assumedly, animal hookworm larvae were unable to complete the life cycle indoors because the floors were usually dry and accidentally dropped animal excrements were rapidly removed.

It remains uncertain whether the infections predominantly took place peridomestically or in public areas, such as parks, as suspected in some outbreak investigations [32–34]. Our findings that the presence of faeces on the compound was an independent risk factor and that playing football on improvised playgrounds was not an independent risk factor indicate that peridomestic transmission is important.

This study shows for the first time that low income and poverty-related living conditions are crucial risk factors for HrCLM. Hitherto, a low family income has been identified as a risk factor but didn´t reach statistical significance in the mulitvariate analysis. The concept of an asset index as a long-term indicator of the socio-economic status of the household has never been applied in earlier studies [8,23].

Even within a poor population, as in the community of Nova Vitoria, the relative level of poverty predicted the risk of acquiring HrCLM. A household income of one minimum wage or less was associated with a high risk of acquiring HrCLM. Also, a low wealth score was an independent risk factor. Hence, the poorest of the poor are the most vulnerable part of the population, which corroborates our hypothesis that occurrence of HrCLM is a proxy of the economic situation in a country [35]. Many neglected tropical diseases are considered to be associated with poverty [36,37] but HrCLM is particular in the sense that it affects the poorest of the poor.

### Policy recommendations

In contrast to other soil-transmitted helminths, HrCLM has a pure animal reservoir and thus treating the human population cannot influence the incidence of HrCLM. Veterinary anthelmintic therapy can be effective [38] but is hard to realise in areas lacking basic infrastructure even for human health. Therefore, disease control strategies have to point towards improvement of living conditions, environmental factors and protective behaviour. Preventing access of cats and dogs to playgrounds and informing the public about pet-associated health risks and protective shoewear will be essential to reduce the parasite burden in humans as long as infrastructure remains precarious [32–34,39,40].

### Limitations

For safety reasons Nova Vitoria could only be visited during daylight hours. Thus, there may have been a selection bias in favour of women and children staying at home versus adult males being at work. By means of an exhaustive sampling strategy, we still obtained a high participation and a representative sample of the daytime population. We have no reasons to believe that study participants with missing data differed from those without missing data and hence any missing observation reduced statistical power but is unlikely to have biased the results [41].

Confusion of HrCLM with other conditions that present as a creeping skin eruption such as gnathostoma, *Strongyloides stercoralis* (larva currens), fly maggots (migratory myiasis) and scabies is theoretically possible [1,6,24]. However, a slightly elevated linear or serpiginous track and the slow velocity of progression with several millimetres to few centimetres per day are pathognonomic [6,42]. We therefore assume that all participants were correctly diagnosed.

### Conclusion

The study revealed the highest prevalence of HrCLM in a representative population sample known to date and showed transmission in peridomestic areas. We could prove that HrCLM is a disease of the poorest of the poor. It is therefore plausible that for elimination of HrCLM as a public health threat, it is necessary to improve the living conditions.

## Supporting Information

S1 Checklist STROBE checklist. (DOCX)

S1 Database Study database. (SAV)

## References

[1] Heukelbach J, Feldmeier H. Epidemiological and clinical characteristics of hookworm-related cutaneous larva migrans. Lancet Infect Dis. 2008;8: 302–309. 10.1016/S1473-3099(08)70098-7 18471775

[2] Beaver PC. Larva migrans. Exp Parasitol. 1956;5: 587–621. 13375685 10.1016/0014-4894(56)90032-7

[3] Fulleborn F. Epidemiological observations on hookworm infection. Br Med J. 1929;1: 755–759. 20774636 PMC2451097

[4] Elliot DL, Tolle SW, Goldberg L, Miller JB. Pet-associated illness. N Engl J Med. 1985;313: 985–995. 10.1056/NEJM198510173131605 3900726

[5] Granzer M, Haas W. Host-finding and host recognition of infective Ancylostoma caninum larvae. Int J Parasitol. 1991;21: 429–440. 1917283 10.1016/0020-7519(91)90100-l

[6] Caumes E, Danis M. From creeping eruption to hookworm-related cutaneous larva migrans. Lancet Infect Dis. 2004;4: 659–660. 10.1016/S1473-3099(04)01178-8 15522674

[7] Schuster A, Lesshafft H, Talhari S, Guedes de Oliveira S, Ignatius R, Feldmeier H. Life Quality Impairment Caused by Hookworm-Related Cutaneous Larva Migrans in Resource-Poor Communities in Manaus, Brazil. PLoS Negl Trop Dis. 2011;5: e1355 10.1371/journal.pntd.0001355 22087341 PMC3210737

[8] Heukelbach J, Wilcke T, Meier A, Sabóia Moura RC, Feldmeier H. A longitudinal study on cutaneous larva migrans in an impoverished Brazilian township. Travel Med Infect Dis. 2003;1: 213–218. 10.1016/j.tmaid.2003.10.003 17291920

[9] Schuster A, Lesshafft H, Reichert F, Talhari S, de Oliveira SG, Ignatius R, et al Hookworm-related cutaneous larva migrans in northern Brazil: resolution of clinical pathology after a single dose of ivermectin. Clin Infect Dis. 2013;57: 1155–1157. 10.1093/cid/cit440 23811416

[10] Lesshafft H, Schuster A, Reichert F, Talhari S, Ignatius R, Feldmeier H. Knowledge, attitudes, perceptions, and practices regarding cutaneous larva migrans in deprived communities in Manaus, Brazil. J Infect Dev Ctries. 2012;6: 422–429. 22610709 10.3855/jidc.2122

[11] Hochedez P, Caumes E. Hookworm-related cutaneous larva migrans. J Travel Med. 2007;14: 326–333. 10.1111/j.1708-8305.2007.00148.x 17883464

[12] Jelinek T, Maiwald H, Nothdurft HD, Löscher T. Cutaneous larva migrans in travelers: synopsis of histories, symptoms, and treatment of 98 patients. Clin Infect Dis. 1994;19: 1062–1066. 7534125 10.1093/clinids/19.6.1062

[13] Davies HD, Sakuls P, Keystone JS. Creeping eruption. A review of clinical presentation and management of 60 cases presenting to a tropical disease unit. Arch Dermatol. 1993;129: 588–591. 8481019 10.1001/archderm.129.5.588

[14] Wilson ME, Chen LH, Han PV, Keystone JS, Cramer JP, Segurado A, et al Illness in Travelers Returned From Brazil: The GeoSentinel Experience and Implications for the 2014 FIFA World Cup and the 2016 Summer Olympics. Clin Infect Dis. 2014; 10.1093/cid/ciu122 PMC7112384 24585698

[15] Tamminga N, Bierman WFW, de Vries PJ. Cutaneous Larva Migrans Acquired in Brittany, France. Emerg Infect Dis. 2009;15: 1856–1858. 10.3201/eid1511.090261 19891887 PMC2857223

[16] Kienast A, Bialek R, Hoeger PH. Cutaneous larva migrans in northern Germany. Eur J Pediatr. 2007;166: 1183–1185. 10.1007/s00431-006-0364-0 17216216

[17] Diba VC, Whitty CJM, Green T. Cutaneous larva migrans acquired in Britain. Clin Exp Dermatol. 2004;29: 555–556. 10.1111/j.1365-2230.2004.01592.x 15347353

[18] Galanti B, Fusco FM, Nardiello S. Outbreak of cutaneous larva migrans in Naples, southern Italy. Trans R Soc Trop Med Hyg. 2002;96: 491–492. 12474474 10.1016/s0035-9203(02)90415-3

[19] Müller-Stöver I, Richter J, Häussinger D. [Cutaneous larva migrans (creeping eruption) acquired in Germany]. Dtsch Med Wochenschr. 2010;135: 859–861. 10.1055/s-0030-1253669 20408105

[20] Veraldi S, Persico MC, Francia C, La Vela V. Appearance of a reservoir of hookworm-related cutaneous larva migrans in Brittany? G Ital Dermatol Venereol. 2012;147: 649–652. 23149711

[21] Heukelbach J, Wilcke T, Feldmeier H. Cutaneous larva migrans (creeping eruption) in an urban slum in Brazil. Int J Dermatol. 2004;43: 511–515. 10.1111/j.1365-4632.2004.02152.x 15230890

[22] Jackson A, Heukelbach J, Calheiros CML, Soares V de L, Harms G, Feldmeier H. A study in a community in Brazil in which cutaneous larva migrans is endemic. Clin Infect Dis. 2006;43: e13–18. 10.1086/505221 16779735

[23] Heukelbach J, Jackson A, Ariza L, Feldmeier H. Prevalence and risk factors of hookworm-related cutaneous larva migrans in a rural community in Brazil. Ann Trop Med Parasitol. 2008;102: 53–61. 10.1179/136485908X252205 18186978

[24] Caumes E. It’s time to distinguish the sign “creeping eruption” from the syndrome “cutaneous larva migrans.” Dermatology (Basel). 2006;213: 179–181. 10.1159/000095032 17033164

[25] Vyas S, Kumaranayake L. Constructing socio-economic status indices: how to use principal components analysis. Health Policy Plan. 2006;21: 459–468. 10.1093/heapol/czl029 17030551

[26] World Health Organization. Helminth control in school-age children: a guide for managers of control programmes [Internet] 2nd ed. Geneva: World Health Organization; 2011 Available: http://whqlibdoc.who.int/publications/2011/9789241548267_eng.pdf

[27] Bouchaud O, Houzé S, Schiemann R, Durand R, Ralaimazava P, Ruggeri C, et al Cutaneous Larva Migrans in Travelers: A Prospective Study, with Assessment of Therapy with Ivermectin. Clin Infect Dis. 2000;31: 493–498. 10.1086/313942 10987711

[28] Blackwell V, Vega-Lopez F. Cutaneous larva migrans: clinical features and management of 44 cases presenting in the returning traveller. Br J Dermatol. 2001;145: 434–437. 11531833 10.1046/j.1365-2133.2001.04406.x

[29] Tremblay A, MacLean JD, Gyorkos T, Macpherson DW. Outbreak of cutaneous larva migrans in a group of travellers. Trop Med Int Health. 2000;5: 330–334. 10886795 10.1046/j.1365-3156.2000.00557.x

[30] Kannathasan S, Murugananthan A, Rajeshkannan N, de Silva NR. Cutaneous larva migrans among devotees of the Nallur temple in Jaffna, Sri Lanka. PLoS ONE. 2012;7: e30516 10.1371/journal.pone.0030516 22295089 PMC3266239

[31] Mandell GL, Dolin R, Bennett JE. Mandell, Douglas, and Bennett’s Principles and Practice of Infectious Diseases [Internet]. Philadelphia: Churchill Livingstone Elsevier; 2010 Available: http://search.ebscohost.com/login.aspx?direct=true&db=nlebk&AN=458761&site=ehost-live

[32] Araújo FR, Araújo CP, Werneck MR, Górski A. Cutaneous larva migrans in children of a school, Brazil. Revista de Saúde Pública. 2000;34: 84–85. 10.1590/S0034-89102000000100015 10769366

[33] Santarém VA, Giuffrida R, Zanin GA. [Cutaneous larva migrans: reports of pediatric cases and contamination by Ancylostoma spp larvae in public parks in Taciba, São Paulo State]. Rev Soc Bras Med Trop. 2004;37: 179–181. 15094907 10.1590/s0037-86822004000200014

[34] Centers for Disease Control and Prevention (CDC). Outbreak of cutaneous larva migrans at a children’s camp—Miami, Florida, 2006. MMWR Morb Mortal Wkly Rep. 2007;56: 1285–1287. 18075486

[35] Feldmeier H, Krantz I. A way of measuring poverty that could further a change for the better. Bull World Health Organ. 2008;86: A. 10.2471/BLT.07.050294 PMC2647474 18568259

[36] Hotez PJ, Fenwick A, Savioli L, Molyneux DH. Rescuing the bottom billion through control of neglected tropical diseases. Lancet. 2009;373: 1570–1575. 10.1016/S0140-6736(09)60233-6 19410718

[37] Alvar J, Yactayo S, Bern C. Leishmaniasis and poverty. Trends Parasitol. 2006;22: 552–557. 10.1016/j.pt.2006.09.004 17023215

[38] Kannathasan S, Murugananthan A, Rajeshkannan N, de Silva NR. A Simple Intervention to Prevent Cutaneous Larva Migrans among Devotees of the Nallur Temple in Jaffna, Sri Lanka. PLoS ONE. 2013;8: e61816 10.1371/journal.pone.0061816 23613943 PMC3629127

[39] Marques JP, Guimarães C de R, Boas AV, Carnaúba PU, Moraes J de. Contamination of public parks and squares from Guarulhos (São Paulo State, Brazil) by Toxocara spp. and Ancylostoma spp. Revista do Instituto de Medicina Tropical de São Paulo. 2012;54: 267–271. 10.1590/S0036-46652012000500006 22983290

[40] Santarém Vamilton Alvares, Rubinsky-Elefant Guita, Ferreira Marcelo Urbano. Soil-Transmitted Helminthic Zoonoses in Humans and Associated Risk Factors. Soil Contamination. 2011 Available: http://www.intechopen.com/books/soil-contamination/soil-transmitted-helminthic-zoonoses-in-humans-and-associated-risk-factors

[41] White IR, Carlin JB. Bias and efficiency of multiple imputation compared with complete-case analysis for missing covariate values. Stat Med. 2010;29: 2920–2931. 10.1002/sim.3944 20842622

[42] Sunderkötter C, von Stebut E, Schöfer H, Mempel M, Reinel D, Wolf G, et al S1 guideline diagnosis and therapy of cutaneous larva migrans (creeping disease). JDDG: Journal der Deutschen Dermatologischen Gesellschaft. 2014;12: 86–91. 10.1111/ddg.12250 24393321

