# Supplementary material for: Prevalence and Risk Factors of Hookworm-Related Cutaneous Larva Migrans (HrCLM) in a Resource-Poor Community in Manaus, Brazil
Source: PLoS Negl Trop Dis. 2016 Mar 24;10(3):e0004514. doi: 10.1371/journal.pntd.0004514 (PMC4807001; doi:10.1371/journal.pntd.0004514)
Supplement: S1 Checklist — (DOCX) [file pntd.0004514.s001.docx]

STROBE Statement—checklist of items that should be included in reports of observational studies

|  | Item No. | Recommendation | Page  No. | Relevant text from manuscript |
| --- | --- | --- | --- | --- |
| **Title and abstract** | 1 | (*a*) Indicate the study’s design with a commonly used term in the title or the abstract | 2 | We conducted a cross-sectional study |
|  |  | (*b*) Provide in the abstract an informative and balanced summary of what was done and what was found | 2 | HrCLMwas diagnosed in 8.2% (95% CI, 6.3-10.1%) of the study population (N=806) with a peak prevalence of 18.2% (95% CI, 9.3-27.1%) in children aged 10-14. Most of the tracks (62.4%) were located on the feet, and 10.6% were superinfected. HrCLM was associated independently with age under 15, male sex, presence of animal faeces on the compound, walking barefoot on sandy ground and poverty. |
| Introduction | | | |  |
| Background/rationale | 2 | Explain the scientific background and rationale for the investigation being reported | 4 | In semi-arid north-eastern Brazil, prevalence [...]. No population based data exists for other endemic areas. |
| Objectives | 3 | State specific objectives, including any prespecified hypotheses | 4 | In order to investigate the epidemiology of HrCLM in Amazonia and to develop sustainable means of control, in a first step we determined prevalence and risk factors in a resource-poor community in the outskirts of Manaus. |
| Methods | | | |  |
| Study design | 4 | Present key elements of study design early in the paper | 5 | See setting |
| Setting | 5 | Describe the setting, locations, and relevant dates, including periods of recruitment, exposure, follow-up, and data collection | 5  6 | The study was conducted in Manaus, capital of Amazonas State, North Brazil [...] The study area is part of Nova Vitoria, a resource-poor neighbourhood at the outskirts of Manaus. The boundaries of the study area are defined on three sides by an *igarapé*, a small affluent of the Amazon River. On the fourth side a paved road separates the study area from Grande Vitoria, another resource-poor community. The study area is characterized by unpaved roads, absence of public health facilities, [...] we conducted a cross-sectional study in Nova Vitoria in April 2009, at the end of the rainy season. First, a census of all households and inhabitants was performed. During a door-to-door survey, households were GPS-mapped and environmental, socio-economic and behaviour-related risk factors were documented using a pre-tested, structured questionnaire.  All participants were examined clinically for HrCLM. |
| Participants | 6 | (*a*) *Cohort study*—Give the eligibility criteria, and the sources and methods of selection of participants. Describe methods of follow-up  *Case-control study*—Give the eligibility criteria, and the sources and methods of case ascertainment and control selection. Give the rationale for the choice of cases and controls  *Cross-sectional study*—Give the eligibility criteria, and the sources and methods of selection of participants | 5 | Inclusion criteria were residency in the study area for more than two months and provision of an informed, written consent. |
|  |  | (*b*) *Cohort study*—For matched studies, give matching criteria and number of exposed and unexposed  *Case-control study*—For matched studies, give matching criteria and the number of controls per case |  | No matching |
| Variables | 7 | Clearly define all outcomes, exposures, predictors, potential confounders, and effect modifiers. Give diagnostic criteria, if applicable | 6 | HrCLM was diagnosed clinically by two investigators (DP and FR) when the characteristic slow-moving, elevated linear or serpiginous tracks were present. Lesions were counted and the appearance and location of the tracks were documented. Each track was defined as a single lesion. Bacterial superinfection was diagnosed when pustules or suppuration were visible. |
| Data sources/ measurement | 8* | For each variable of interest, give sources of data and details of methods of assessment (measurement). Describe comparability of assessment methods if there is more than one group | *5*  *6* | Environmental, socio-economic and behaviour-related risk factors were documented using a pre-tested, structured questionnaire.  All participants were examined clinically for HrCLM. |
| Bias | 9 | Describe any efforts to address potential sources of bias | 6 | HrCLM was diagnosed clinically by two investigators (DP and FR) [...] Each affected inhabitant of Nova Vitoria was offered free treatment independently of the participation in the study.  [to minimize inter-observer and selection bias] |
| Study size | 10 | Explain how the study size was arrived at | 7 | According to the census 412 households existed in the study area, 127 of which were found without a resident present. Of the remaining 285 households, 5 (2%) did not match the inclusion criteria and 18 (6%) refused to participate. The remaining 262 households (92%) were inhabited by a total of 1104 people out of whom 806 (73%) were present during sampling and were included in the study. |

Continued on next page

| Quantitative variables | 11 | Explain how quantitative variables were handled in the analyses. If applicable, describe which groupings were chosen and why | 6-7 | An asset index was formed using principal component analysis (PCA) to categorize households according to socio-economic status. First, a set of assets that reflect wealth were identified. From this set of assets, we selected items with a high inequity in distribution among the households and a high eigenvalue. Included assets were presence of a car, television, fridge, type of house construction, legal connection to electricity and monthly mobile phone costs. Using these assets, an index (“wealth score”) was built based on the respective value of each item in the PCA. Households were ranked and divided into tertiles representing a high, intermediate or low socio-economic status. Income was categorized into three categories with the official minimum wage ([R$](http://en.wikipedia.org/wiki/Brazilian_real) 465 per month in 2009) as a reference.  A knowledge score was derived out of six questions concerning the etiology of HrCLM. Every correct answer added one point to the score. The knowledge score values were categorized in tertiles representing households with little knowledge (0-3 correct answers), moderate knowledge (4 correct answers) and high knowledge (5-6 correct answers). Age groups were formed similar to previous population-based studies on HrCLM to allow comparison of the results |
| --- | --- | --- | --- | --- |
| Statistical methods | 12 | (*a*) Describe all statistical methods, including those used to control for confounding | 7 | For bivariable risk factor analysis, odds ratios (OR) were calculated together with 95% confidence intervals (95% CI). Statistical analysis consisted of χ²-test or Fisher-exact-test to compare relative frequencies and logistic regression for non-binary variables.  For multivariable risk factor analysis, all variables that showed weak evidence of an association with HrCLM (p<0.1) were entered into a stepwise logistic regression. We observed standard errors and 95% CI to identify multicollinearity and removed variables where necessary. A random effects model was used to control for clustering on household level. |
|  |  | (*b*) Describe any methods used to examine subgroups and interactions |  | No subgroup analysis besides Prevalence by age group and sex |
|  |  | (*c*) Explain how missing data were addressed | 6 | Missing data were assumed to be missing at random and flagged up in the analysis. |
|  |  | (*d*) *Cohort study*—If applicable, explain how loss to follow-up was addressed  *Case-control study*—If applicable, explain how matching of cases and controls was addressed  *Cross-sectional study*—If applicable, describe analytical methods taking account of sampling strategy | 7 | A random effects model was used to control for clustering on household level. |
|  |  | (*e*) Describe any sensitivity analyses |  |  |
| Results | | | | |
| Participants | 13* | (a) Report numbers of individuals at each stage of study—eg numbers potentially eligible, examined for eligibility, confirmed eligible, included in the study, completing follow-up, and analysed |  | See item No. 10 |
|  |  | (b) Give reasons for non-participation at each stage |  | See item No. 10 |
|  |  | (c) Consider use of a flow diagram |  |  |
| Descriptive data | 14* | (a) Give characteristics of study participants (eg demographic, clinical, social) and information on exposures and potential confounders | 8-10 | Seventy-eight per cent of the adults were unemployed or working in the informal sector. Fifty-eight per cent of the households had one minimum wage ([R$](http://en.wikipedia.org/wiki/Brazilian_real) 465 per month) or less at their disposition. The proportion of illiteracy in adults was at least 27%. Only 11.5% of the households had been visited by a community health worker within the last 12 months. Thirty-one per cent of the households stated that at least one case of HrCLM had occurred in household members within the last 12 months. [...]  The median age was 13 years (range 0-72). The majority of the participants were females (59.3%). Sixty-six persons (8.2%; 95% CI, 6.3-10.1%) had HrCLM with a total of 117 lesions. Clinical characteristics of the infected study participants are presented in Table 2. Children aged 10-14 had the highest prevalence (18.2%; 95% CI, 9.3-27.1%; Figure 1). In all age groups of children, boys were significantly more affected than girls (p<0.001). The feet were the most common localisation of HrCLM.  Previous episodes of HrCLM were remembered of 18.7% of the participants. Following anamnestic information 39.7% had suffered of pediculosis capitis, 26.8% of tungiasis and 5.7% of scabies in the past year.  See also table 1 and 2. |
|  |  | (b) Indicate number of participants with missing data for each variable of interest |  | Indicated in the tables |
|  |  | (c) *Cohort study*—Summarise follow-up time (eg, average and total amount) |  |  |
| Outcome data | 15* | *Cohort study*—Report numbers of outcome events or summary measures over time |  |  |
|  |  | *Case-control study—*Report numbers in each exposure category, or summary measures of exposure |  |  |
|  |  | *Cross-sectional study—*Report numbers of outcome events or summary measures | 11-13 | Table 3 |
| Main results | 16 | (*a*) Give unadjusted estimates and, if applicable, confounder-adjusted estimates and their precision (eg, 95% confidence interval). Make clear which confounders were adjusted for and why they were included |  | Table 3 and 4.  Bivariable risk factor analysis showed that male sex, age younger than 15, low family income, a low wealth score, playing football, practicing sport barefoot and presence of animal faeces on the compound were significantly associated with a high risk of HrCLM (Table 3). Those who reported to have had HrCLM in the last year had a significantly higher risk to be diagnosed with HrCLM in the cross-sectional study (OR=15; 95% CI, 8.5-26.7). The highest risk was associated with the habit of always walking barefoot on sandy ground or soil (OR=23.4; 95% CI, 8.0-68.6). |
|  |  | (*b*) Report category boundaries when continuous variables were categorized | 7 | Age groups were formed similar to previous population-based studies on HrCLM to allow comparison of the results |
|  |  | (*c*) If relevant, consider translating estimates of relative risk into absolute risk for a meaningful time period |  |  |

Continued on next page

| Other analyses | 17 | Report other analyses done—eg analyses of subgroups and interactions, and sensitivity analyses |  |  |
| --- | --- | --- | --- | --- |
| Discussion | | | | |
| Key results | 18 | Summarise key results with reference to study objectives | 15-17 | Clinical features were similar to those reported by others. Most of the tracks (62.4%) were located on the feet, which reflects the fact that many people walked barefoot. [...] The percentage of superinfected tracks was 10.6%.[...]  The overall prevalence of 8.2% (95% CI 6.3-10.1%) found in this study is the highest ever documented in a population-based study. [...]  For the first time we could show that the odds differed by the frequency protective footwear was used. Participants who always used shoes ran a lower risk of acquiring HrCLM than those wearing shoes sometimes[...]  This study shows for the first time that low income and poverty-related living conditions are crucial risk factors for HrCLM.[...] |
| Limitations | 19 | Discuss limitations of the study, taking into account sources of potential bias or imprecision. Discuss both direction and magnitude of any potential bias | 18 | For safety reasons Nova Vitoria could only be visited during daylight hours. Thus, there may have been a selection bias in favour of women and children staying at home versus adult males being at work. By means of an exhaustive sampling strategy, we still obtained a high participation and a representative sample of the daytime population. We have no reasons to believe that study participants with missing data differed from those without missing data and hence any missing observation reduced statistical power but is unlikely to have biased the results.  Confusion of HrCLM with other conditions that present as a creeping skin eruption such as gnathostoma, *Strongyloides stercoralis* (larva currens), fly maggots (migratory myiasis) and scabies is theoretically possible. However, a slightly elevated linear or serpiginous track and the slow velocity of progression with several millimetres to few centimetres per day are pathognonomic. We therefore assume that all participants were correctly diagnosed. |
| Interpretation | 20 | Give a cautious overall interpretation of results considering objectives, limitations, multiplicity of analyses, results from similar studies, and other relevant evidence | 15-17 | Clinical features were similar to those reported by others. Most of the tracks (62.4%) were located on the feet, which reflects the fact that many people walked barefoot [...]  Unhygienic living conditions and practices as well as limited access to healthcare may explain the higher proportion of superinfected HrCLM in our study than usually seen in travellers [...]  Previous population-based studies in Northeast Brazil showed an overall prevalence between 0.2% and 4.4% during the dry and the raining season, respectively [...] Outside Brazil only one prevalence study has been conducted on devotees of a temple in Sri Lanka. Fifty-eight per cent of the devotees were found to have HrCLM; however, it is doubtful whether this finding reflects the true overall prevalence in that area since the participants were examined after a special religious ritual increasing the odds for exposure. [...]  The extremely high prevalence found in our study indicates excellent conditions for the completion of the off-host cycle of animal hookworm in Nova Vitoria. First, many stray dogs and cats roam in the community and act as animal reservoirs. There is no public veterinary service at all and pets are not treated against intestinal helminths. Animal faeces were present on 11.8% of all compounds, and faecal material littered many public areas. Second, hookworm eggs require an environment that protects them from desiccation to evolve into infective third stage larvae. Manaus is located in the middle of the Amazon basin. The precipitation in the month preceding the study was around 230 mm with 20 days of rain (International Institute of Meteorology of Brazil (INMET)). All streets and most of the compounds in Nova Vitoria were unpaved and became muddy after heavy rainfall. Furthermore, the average temperature never falls below 25° C. This means that the environmental conditions are exceptionally favourable for the propagation of animal hookworm larvae. And third, risky behaviour with prolonged contact to contaminated soil was frequent. Many children did not go to school but roamed through the streets and compounds the whole day, the majority walking barefoot at least part of the time. [...]  The multivariable model showed a complex pattern of risk factors with walking barefoot on sandy soil being most significant. This corroborates our previous findings from a semi-arid area of Brazil, where the lacking use of footwear was an independent risk factor [...]  Hitherto, a low family income has been identified as a risk factor but didn´t reach statistical significance in the mulitvariate analysis. [...] Even within a poor population, as in the community of Nova Vitoria, the relative level of poverty predicted the risk of acquiring HrCLM. A household income of one minimum wage or less was associated with a high risk of acquiring HrCLM. Also, a low wealth score was an independent risk factor. Hence, the poorest of the poor are the most vulnerable part of the population |
| Generalisability | 21 | Discuss the generalisability (external validity) of the study results | 5 | The study area is characterized by unpaved roads, absence of public health facilities, kindergartens or public schools. There was no sewage disposal system at the time of the study. Electricity was available but only half of the households were legally connected to the grid; the other half used hand-made wire connections. Drinking water was distributed via rubber hoses, which often flooded the streets. Many cats and dogs strayed around in the streets and gardens. Children usually played on the compound of the house, in the streets or on improvised football fields. Hence, the study area was representative for the innumerable poor neighbourhoods at the periphery of Manaus. |
| Other information | |  | | |
| Funding | 22 | Give the source of funding and the role of the funders for the present study and, if applicable, for the original study on which the present article is based |  | As required by PLOS submission guidelines, the funding statement is only part of additional information requested at submission:  The Brazilian Coordination for the Improvement of Higher Education Personell (CAPES) partially supported the study. The German Academic Exchange Service (DAAD) provided travel grants to FR and AS).  The funders had no role in study design, data collection and analysis, decision to publish, or preparation of the manuscript.  The Fundação de Medicina Tropical do Amazonas (FMT-AM) provided infrastructure and helped to identify the study area. |

*Give information separately for cases and controls in case-control studies and, if applicable, for exposed and unexposed groups in cohort and cross-sectional studies.

**Note:** An Explanation and Elaboration article discusses each checklist item and gives methodological background and published examples of transparent reporting. The STROBE checklist is best used in conjunction with this article (freely available on the Web sites of PLoS Medicine at http://www.plosmedicine.org/, Annals of Internal Medicine at http://www.annals.org/, and Epidemiology at http://www.epidem.com/). Information on the STROBE Initiative is available at www.strobe-statement.org.
